# New Approaches to the Biology of Stomatal Guard Cells

**DOI:** 10.1093/pcp/pct145

**Published:** 2013-11-07

**Authors:** Juntaro Negi, Mimi Hashimoto-Sugimoto, Kensuke Kusumi, Koh Iba

**Affiliations:** ^1^Department of Biology, Faculty of Sciences, Kyushu University, Fukuoka, 812-8581 Japan; ^2^These authors contributed equally to this work

**Keywords:** *Arabidopsis*, Carbon dioxide, HT1 kinase, Thermal imaging, SLAC1 anion channel, Stomata

## Abstract

CO_2_ acts as an environmental signal that regulates stomatal movements. High CO_2_ concentrations reduce stomatal aperture, whereas low concentrations trigger stomatal opening. In contrast to our advanced understanding of light and drought stress responses in guard cells, the molecular mechanisms underlying stomatal CO_2_ sensing and signaling are largely unknown. Leaf temperature provides a convenient indicator of transpiration, and can be used to detect mutants with altered stomatal control. To identify genes that function in CO_2_ responses in guard cells, CO_2_-insensitive mutants were isolated through high-throughput leaf thermal imaging. The isolated mutants are categorized into three groups according to their phenotypes: (i) impaired in stomatal opening under low CO_2_ concentrations; (ii) impaired in stomatal closing under high CO_2_ concentrations; and (iii) impaired in stomatal development. Characterization of these mutants has begun to yield insights into the mechanisms of stomatal CO_2_ responses. In this review, we summarize the current status of the field and discuss future prospects.

## Introduction

**Fig. 1:**
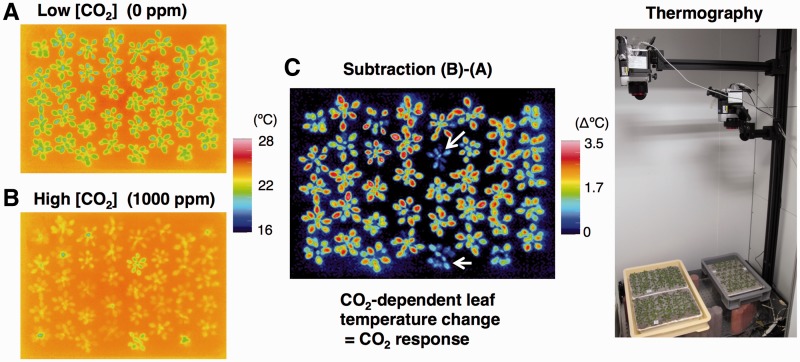
Strategy for the isolation of Arabidopsis mutants using altered CO_2_ responses indicated by leaf temperature. (A) Thermal image of plants subjected to low [CO_2_]. Plants exhibit lower leaf temperatures. (B) Thermal image of plants subjected to high [CO_2_]. Plants exhibit higher leaf temperatures. (C) Image of leaf temperature change in response to CO_2_. This image displays the subtraction of image (A) from image (B). The arrow indicates a mutant plant showing impaired CO_2_-dependent leaf temperature change .

Stomata are pores in the plant epidermis that function as gateways linking the intercellular gas spaces to the external environment. Two guard cells surround each stomatal pore, and changes in turgor pressure of the guard cells regulate the size of the pore aperture. An increase in guard cell turgor results in stomatal opening, whereas a reduction in turgor leads to stomatal closure (Willmer and Fricker 1996). Stomata can control CO_2_ assimilation and limit excessive water loss by optimizing the aperture in response to the changing external environment, and by integrating a variety of stimuli, for example light, ABA and CO_2_ (MacRobbie 1998, Blatt 2000, Hetherington and Woodward 2003, Shimazaki et al. 2007, Kim et al. 2010). The CO_2_ concentration ([CO_2_]) inside the leaves changes as a result of photosynthesis and transpiration; low [CO_2_] induces stomatal opening while high [CO_2_] induces stomatal closure (Hanstein et al. 2001). Furthermore, the continuing rise in atmospheric [CO_2_] is predicted to interfere with the regulation of stomatal conductance (rate of water vapor exiting from the stomata) (Morison 1987, Medlyn et al. 2001), and to have diverse and dramatic effects on the productivity of agriculture, the plant ecosystem and the global climate (Hetherington and Woodward 2003, Ainsworth and Rogers 2007, Keenan et al. 2013). Biochemical and electrophysiological studies have contributed substantial insights regarding ion transporters that mediate transmembrane ion (e.g. K^+^, H^+^, Cl^−^, malate^−^ and Ca^2+^) fluxes during stomatal CO_2_ responses (Hedrich and Marten 1993, Webb and Hetherington 1997, Assmann 1999, Blatt 2000, Schroeder et al. 2001). However, the mechanisms and components underlying the responses are still debated. Leaf temperature provides physiological information regarding stomatal movements and transpiration (Hashimoto et al. 1984, Jones 1999). Making use of thermal imaging, genetic screens of mutants with altered stomatal responses to drought (Raskin and Ladyman 1988, Merlot et al. 2002, Merlot et al. 2007), low humidity (Xie et al. 2006), [CO_2_] (Hashimoto et al. 2006, Negi et al. 2008, Hashimoto-Sugimoto et al. 2013, Negi et al. 2013: see also Fig. 1) and blue light (Takemiya et al. 2013) have been performed (Table 1). Here, we will introduce four novel components of stomatal function that were identified through the genetic and functional analysis of CO_2_ response mutants.

**Table 1:** List of Arabidopsis mutants isolated by thermography

Condition of mutant screening	Mutant name	Mutation locus	Gene description	References
Drought stress/humidity	*aba2*	AT1G52340	Enzyme involed in ABA biosynthesis	Merlot et al. (2002); Xie et al. (2006)
	*ost1*	AT4G33950	SNF1-related protein kinases (SnRK2e)	Merlot et al. (2002); Mustilli et al. (2002); Xie et al. (2006)
	*ost2*	AT2G18960	Plasma membrane proton ATPase (AHA1)	Merlot et al. (2007)
CO_2_	*ht1*	AT1G62400	Serine/threonine-protein kinase	Hashimoto et al. (2006)
	*slac1*	AT1G12480	S-type anion channel	Negi et al. (2008)
	*scap1*	AT5G65590	Dof-type transcription factor (AtDof5.8)	Negi et al. (2013)
	*patrol1*	AT5G06970	Munc13-like protein	Hashimoto-Sugimoto et al. (2013)
Blue light	*blus1*	AT4G14480	Serine/threonine-protein kinase	Takemiya et al. (2013)

## HT1 Protein Kinase and Carbonic Anhydrase Function in CO_2_-Specific Signaling Pathways

**Fig. 2:**
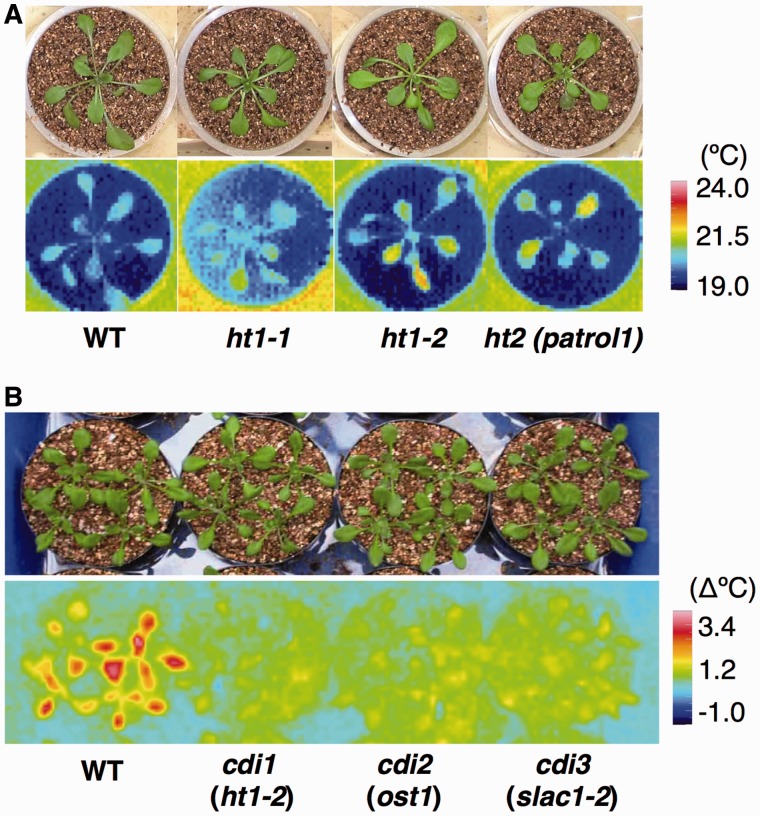
Phenotype of CO_2_ response mutants. (A) Thermal image of plants subjected to low [CO_2_]. *ht* (*high leaf temperature*) mutants exhibit higher leaf temperatures than wild-type (WT) plants. (B) Subtractive thermal image of plants when transfered from high to low [CO_2_]. *cdi* (*carbon dioxide insensitive*) mutants show a CO_2_-insensitive phenotype.

The first Arabidopsis mutant with impaired CO_2_ response isolated by thermal imaging was *ht1* (*high leaf temperature 1*; *ht1-1* and *ht1-2*) (Hashimoto et al. 2006) (Fig. 2). Plants carrying the strong *ht1-2* allele are completely impaired in stomatal CO_2_ responses; however, they show functional responses to blue light, fusicoccin (FC) and ABA (Hashimoto et al. 2006). This indicates that HT1 is a central regulator of stomatal CO_2_ signaling. Further analyses demonstrated that HT1 is a protein kinase expressed mainly in guard cells, and that the HT1 kinase activities of the mutants, dominant-negative transgenic plants and wild-type (WT) plants corresponded to their ability to perform stomatal responses to CO_2_ (Hashimoto et al. 2006). Evidently, phosphorylation by HT1 kinase is an essential process in CO_2_ signaling.

**Fig. 3:**
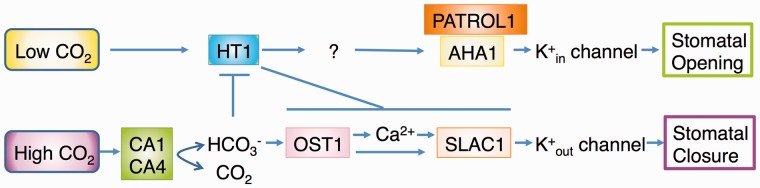
Model of signaling events induced by low and high CO_2_, illustrating the functions of recently identified genes and mechanisms in guard cells. HT1, HIGH LEAF TEMPERATRURE 1; AHA1, ARABIDOPSIS H^+^-ATPase 1; PATROL1, PROTON ATPase TRANSLOCATION CONTROL 1; CA, carbonic anhydrase; OST1, OPEN STOMATA 1; SLAC1, SLOW ANION CHANNEL 1.

A recent study demonstrated that carbonic anhydrases (CAs) can be involved in CO_2_ signaling (Hu et al. 2010). CA is one of the CO_2_-binding proteins, and it catalyzes the reversible reaction CO_2_ + H_2_O ↔ 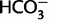 + H^+^. βCA1 and βCA4 are highly expressed in guard cells, and the double mutant *ca1 ca4* shows impaired CO_2_ regulation of stomatal movements, while blue light and ABA responses are not affected (Hu et al. 2010). The *ca1 ca4* plants exhibit increased stomatal densities, while βCA-overexpressing plants show the opposite effect, suggesting that βCA1 and βCA4 function not only in stomatal movement but also in stomatal development (Hu et al. 2010). *ca1 ca4 ht1-2* triple mutants exhibit an impaired response to CO_2_ similarly to *ht1-2* plants, indicating that HT1 is epistatic to βCA1 and βCA4 (Fig. 3). Since stomatal densities of *ht1-2* mutants are normal, HT1 and βCAs may function in independent signaling pathways in stomatal development. High cytoplasmic [CO_2_] together with high bicarbonate concentrations ([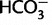]) contributes to the activation of guard cell S (slow activating)-type anion channels (Hu et al. 2010). SLAC1 (SLOW ANION CHANNEL 1), an S-type anion channel, is required for ABA- and Ca^2+^-induced stomatal closure (Vahisalu et al. 2008). Guard cell protoplasts of *slac1* mutants display small anion currents even in the presence of high intracellular [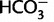] + [CO_2_], demonstrating the important role of SLAC1 (Xue et al. 2011). Elevated intracellular [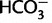], rather than [CO_2_] or [H^+^], mediates the activation of S-type anion currents (Xue et al. 2011). *ht1-2* guard cells show enhanced sensitivity to cytosolic [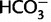] for the activation of S-type anion currents. ABA and ozone induce phosphorylation of SLAC1 channels by an SnRK2-type protein kinase, OST1 (OPEN STOMATA 1) (Geiger et al. 2009, Lee et al. 2009, Vahisalu et al. 2010). *OST1* loss-of-function mutants are impaired in the bicarbonate activation of S-type anion currents, suggesting that OST1 may function at the convergence point of CO_2_ and ABA signaling (Xue et al. 2011) (Fig. 3). In tobacco, mitogen-activated potein kinase (MAPK) pathways are important for stomatal CO_2_ responses, for example *Nt*MPK4-silenced plants are completely insensitive to changes in [CO_2_] but still respond to ABA, and show non-CO_2_-dependent activation of S-type anion channels (Marten et al. 2008). This is similar to HT1 kinase which is a putative MAPKKK (Ichimura et al. 2002). MAPK signaling cascades may affect the OST1 kinase activity in CO_2_ signaling pathways (Fig. 3). ABA-activated OST1 phosphorylates not only SLAC1 but also the 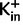 channel KAT1, and this modification reduces the K^+^ transport activity of KAT1 in *Xenopus* oocytes and yeast systems (Sato et al. 2009). This process may also operate in elevated CO_2_ signaling, which would inhibit KAT1 channel activity and lead to stomatal closure.

## SLAC1 is a Major Effector in Stomatal Movements

The regulation of stomatal aperture depends on the transport of ions and organic metabolites across guard cell membranes (Keller et al. 1989, Schroeder and Hagiwara 1989). Malate^2−^ and Cl^−^ efflux from guard cells by means of anion channels mediates membrane depolarization of guard cells (Schroeder and Hagiwara 1989), which in turn is essential for driving K^+^ efflux from guard cells during stomatal closure (MacRobbie 1998, Hetherington 2001, Schroeder et al. 2001). Therefore, anion channels in the plasma membrane (PM) of guard cells were proposed to provide a central control mechanism for stomatal closure. Increased [CO_2_] has been shown to enhance anion channel activity in guard cells of *Vicia faba* (Brearley et al. 1997, Roelfsema et al. 2002, Raschke et al. 2003) and *Nicotiana tabacum* (Marten et al. 2008). Anion channels and their regulatory mechanisms have been characterized primarily using electrophysiological techniques over the last 20 years; however, no genes encoding anion channels involved in stomatal closure were identified until recently (Negi et al. 2008, Saji et al. 2008, Vahisalu et al. 2008).

Negi et al. (2008) used thermography to isolate the Arabidopsis mutant *cdi3* (*carbon dioxide insensitive 3*) that is impaired in CO_2_-dependent leaf temperature change (Fig. 2). *cdi3* mutations abolish CO_2_-, ABA- and darkness-induced stomatal closure. The CDI3 protein is a distant homolog of bacterial and fungal C4-dicarboxylate transporters, and is localized specifically in the PM of guard cells. From these results, it appeared that *CDI3* may encode a long sought subunit of guard cell anion channels. To test this hypothesis, the levels of organic and inorganic ions in guard cell protoplasts of *cdi3* and WT plants were determined. Interestingly, *cdi3* protoplasts showed higher contents of malate and fumarate, compared with WT control protoplasts, whereas succinate levels were not affected. The level of Na^+^ in *cdi3* protoplasts was similar to that of control protoplasts, and *cdi3* protoplasts exhibited a higher content of K^+^ and Cl^−^ (Negi et al. 2008). Parallel to this research, Vahisalu et al. (2008) characterized ozone signaling mutants called *rcd3* (radical induced cell death) in Arabidopsis and identified a mutation in the same gene *CDI3*. Guard cell PM anion channels that mediate anion efflux are classified electrophysiologically as S-type or R (rapid activating)-type anion channels (Schroeder et al. 2001). Patch clamp analyses of *rcd3* mutants showed that S-type anion channel currents were greatly impaired, whereas the R-type anion channel currents were intact in guard cells (Vahisalu et al. 2008). These results supported the idea that CDI3/RCD3 provides or regulates a gate for anion transport, and CDI3/RCD3 was renamed SLAC1 (Negi et al. 2008, Vahisalu et al. 2008).

S-type anion channels in guard cells are activated by phosphorylation (Schmidt et al. 1995). Electrophysiological experiments in *Xenopus* oocytes demonstrated that in the presence of the protein kinase OST1 (Mustilli et al. 2002), SLAC1 generates S-type anion channel activity (Geiger et al. 2009, Lee et al. 2009). These studies proved that SLAC1 is indeed an S-type anion channel, which is activated by OST1-mediated phosphorylation. The three-dimensional structure of the SLAC1 channel has recently been predicted based on homology with the *Haemophilus influenzae* TehA protein (Chen et al. 2010). In both the SLAC1 model and the TehA crystal structure, the channel pore is occluded by the phenyl side chain of a phenylalanine residue. This residue (F450 in SLAC1) is conserved in the entire SLAC1 protein family. Replacing this residue by alanine rendered SLAC1 anion permeable, even in the absence of functional OST1 kinase (Chen et al. 2010). This behavior might indicate that phosphorylation of a cytosolic site, possibly by structural rearrangements, affects the position of the pore-lining F450.

Anion channels are major effectors in stomatal movements and are targeted by a multitude of stimuli, including, CO_2_, ABA, Ca^2+^, methyl jasmonate and elicitors (Kollist et al. 2011, Roelfsema et al. 2012, Kurusu et al. 2013). Recent research focused on the regulatory events that trigger anion channel activation. The calcium-dependent protein kinases CPK21 and CPK23 phosphorylate and activate SLAC1 similarly to OST1 (Geiger et al. 2010). CPK3 and CPK6 were found to participate in the ABA- and Ca^2+^-dependent regulation of guard cell S-type anion channels and stomatal closure (Mori et al. 2006). Recently, CPK6 was shown to activate SLAC1-mediated anion currents strongly in *Xenopus* oocytes and to allow the functional reconstitution of ABA activation of SLAC1 (Brandt et al. 2012). The receptor-like kinase GHR1, mainly localized in the guard cell PM, activates SLAC1 anion currents in *Xenopus* oocytes (Hua et al. 2012). While several regulators involved in the ABA-induced activation of SLAC1 have been identified, the molecular mechanism by which CO_2_ controls SLAC1 activity remains largely unknown. It has been demonstrated that OST1 is a positive regulator of CO_2_-induced stomatal closure and activation of the S-type anion channels in guard cells (Xue et al. 2011). Actually, a new *ost1* allele, *cdi2*, is impaired in CO_2_-dependent leaf temperature change (J. Negi et al. unpublished result: Fig. 2). These data suggest that CO_2_ stimulates OST1, which in turn leads to the activation of SLAC1. The concentration of intracellular free calcium ions ([Ca^2+^]_i_) has been shown to mediate CO_2_ signal transduction in guard cells (Schwartz 1985, Webb et al. 1996, Young et al. 2006). Elevated [Ca^2+^]_i_ in guard cells activates S-type anion channels (Schroeder and Hagiwara 1989). Xue et al. (2011) showed that the bicarbonate activation of S-type anion channels requires elevated [Ca^2+^]_i_. They proposed that CO_2_ enhances the [Ca^2+^]_i_ sensitivity of stomatal closure mechanisms (for a review, see Hubbard et al. 2012) and that CO_2_ activates Ca^2+^-dependent and Ca^2+^-independent signaling pathways to regulate SLAC1 (Fig. 3).

These characteristics of SLAC1 are at least partially conserved in other plant species. Using thermography, Kusumi et al. (2012) isolated SLAC1-deficient mutants in rice with a constitutive low leaf temperature phenotype. SLAC1 deficiency led to an increase in stomatal conductance that paralleled enhanced rates of photosynthesis. These authors further showed that in SLAC1-deficient rice, the ratios of internal [CO_2_] to ambient [CO_2_] (*C*_i_/*C*_a_) increased compared with the WT, whereas there was no significant change in the response of photosynthesis to internal [CO_2_] (*A*/*C*_i_ curves). It seems that in rice the stomatal conductance determines the *C*_i_/*C*_a_ ratio and thereby limits photosynthetic CO_2_ assimilation. These observations suggest a conservation of SLAC1 function among higher plants, and the possibility to develop tools for genetic engineering that improve the productivity and yield of crops.

Various mechanisms have been suggested that couple a rise in [CO_2_] to changes in the activity of PM ion channels. Hedrich et al*.* (1994) found that the apoplastic malate concentration rises in response to high [CO_2_], which can activate R-type anion channels in guard cells. Recently the *AtALMT12*/*QUAC1* gene has been shown to encode an R-type anion channel component in guard cells (Meyer et al. 2010, Sasaki et al. 2010). AtALMT12/QUAC1, a member of the aluminum-activated malate transporter family in Arabidopsis, is highly expressed in guard cells and is targeted to the PM. Plants lacking AtALMT12/QUAC1 are impaired in CO_2_-induced stomatal closure, as well as in ABA responses (Meyer et al. 2010). Electrophysiological studies of loss-of-function mutant guard cells and *Xenopus* oocytes expressing the protein revealed that AtALMT12/QUAC1 represents the malate-sensitive R-type anion channel (Meyer et al. 2010).

The ABC transporter AtABCB14, identified as a malate uptake transporter in the guard cell PM, functions as a negative regulator of CO_2_-induced stomatal closure (Lee et al. 2008). Plants lacking the AtABCB14 transporter exhibited more rapid high CO_2_-induced stomatal closure in comparison with WT controls. However, in isolated epidermal strips that contained guard cells, no difference in stomatal CO_2_ responses was observed between the WT and the *atabcb14* mutant. In contrast, malate-dependent stomatal closure was faster in this mutant and slower in *AtABCB14*-overexpressing plants. This study suggested that AtABCB14 removes extracellular malate which is known to activate anion channels (Hedrich et al. 1994) and, consequently, that part of the CO_2_ response is mediated by malate secreted into the apoplast.

## SCAP1, a Dof Transcription Factor Essential for the Development of Functional Stomata

**Fig. 4:**
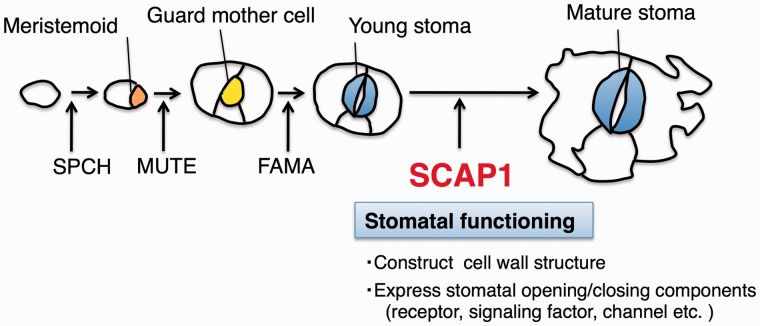
Mechanisms of stomatal development. Three bHLH-type transcription factors (SPCH, MUTE and FAMA) are involved in early stomatal development. A plant-specific transcription factor, SCAP1, directs terminal steps of stomatal development—the functional morphogenesis of kidney-shaped guard cells and the acquisition of specific mechanisms of ion homeostasis including ion channel expression.

Stomata have an elaborate architecture. The differential cell wall thickenings in mature guard cells allow them to alter their shape in response to changes in turgor pressure, which enables guard cells to act as valves (Bergmann and Sack 2007). Young guard cells generate the uneven thickening pattern of the walls. At the same time, they seem to undergo significant gene expression changes to acquire the ability to control ion balance, which is necessary for stomatal movement. Mechanisms of cell fate decision during stomal development in plants resemble those controlling myogenesis and neurogenesis in animals, and basic helix–loop–helix (bHLH)-type transcription factors (SPCH, MUTE and FAMA) are involved in early stomatal development (Pillitteri and Torii 2007, Pillitteri and Torii 2012: see also Fig. 4). However, the mechanisms controlling the functional differentiation of stomata have remained obscure. A recent paper describes the identification of a protein that regulates the maturing and functioning of stomatal guard cells. Negi et al. (2013) have applied thermography to isolate an Arabidopsis mutant, *scap1* (*stomatal carpenter 1*), that develops irregularly shaped guard cells and lacks the ability to control stomatal aperture. *SCAP1* encodes a Dof-type transcription factor (AtDof 5.8) whose expression starts at a late stage of guard cell differentiation. SCAP1 regulates the expression of genes encoding key elements of stomatal functioning and morphogenesis, such as a K^+^ channel protein (Hosy et al. 2003), a MYB60 transcription factor (Cominelli et al. 2005) and pectin methylesterase (Micheli 2001). Furthermore, SCAP1 is required for dimethyl esterification of pectin in guard cell walls and ion homeostasis in guard cells. These results indicate that SCAP1 functions as a transcriptional regulator involved in stomatal maturing and functioning, suggesting the existence of a process that controls completion of the final stage of stomata formation (Fig. 4).

Dof factors are plant-specific transcription factors with function in a variety of physiological contexts (Yanagisawa 2002). Guard cell-specific expression of the K^+^ channel protein gene KST1 and the transcription factor gene *MYB60* is mediated by Dof-binding consensus sequences in their promoter regions (Plesch et al. 2001, Cominelli et al. 2011). Consequently, unidentified Dof factor(s) were proposed to be involved in guard cell-specific gene expression (Galbiati et al. 2008, Yang et al. 2008, Gardner et al. 2009, Cominelli et al. 2011). The *scap1* mutation resulted in decreased expression of MYB60 (Negi et al. 2013). SCAP1 directly binds to the *MYB60* promoter region, which is essential for guard cell-specific expression (Negi et al. 2013). Thus, SCAP1 is one of the Dof factors responsible for the regulation of guard cell-specific gene expression.

Guard cell walls are composed of radially arranged cellulose microfibrils and pectins, and are covered by cuticle (Willmer and Fricker, 1996). Pectins are a complex group of acidic polysaccharides that form a network co-extensive with that of cellulose and hemicelluloses. Pectins may account for up to 30% of the dry weight of a plant cell wall, and guard cells are particularly rich in these polymers (Raschke 1979). Immunolocalization studies showed that guard cell walls contain highly esterified pectins (Majewska-Sawka et al. 2002, Jones et al. 2003). Treatment of epidermal peels with a combination of pectin methylesterase and endopolygalacturonase results in the development of greatly enlarged aperture in response to FC. In contrast, treatment with an arabinase, which hydrolyzes arabinosyl side chains of the pectin rhamnogalacturonan I, inhibits stomatal opening and closure (Jones et al. 2003). Based on these experiments, Jones et al. (2003) suggest that the specific structure of pectins within the guard cell wall can either promote or retard stomatal movements. Stomatal development and maturation might be accompanied by a cell wall modification that renders the ventral cell walls less extensible than the other sides, yet elastic enough so that the ventral walls bend apart to open the stomatal pore. Negi et al. (2013) showed that the demethylesterification of pectins was suppressed in the ventral cell walls of *scap1* guard cells. A subset of stomata in this mutant appeared morphologically abnormal, indicating a disruption of pore morphogenesis. In particular, the ventral cell walls appeared floppy and seemed to adhere to each other in mature stomata. These findings suggest that SCAP1-mediated demethylesterification of pectins enables the unique elasticity of guard cell walls.

The radial array of microtubules that forms during the morphogenesis of guard cells is thought to control the deposition of radial cellulose microfibrils, which determine the final guard cell shape (Zhao and Sack 1999, Lucas et al. 2006). Localized cell wall thickening also influences guard cell shape (Lucas et al. 2006). Nadeau and co-workers have taken a genetic approach to study stomatal morphology. The Arabidopsis mutant *mus* (*mustaches*) is defective in guard cell shape and pore formation (Nadeau and Sack 2002). This mutant has a more severe defect in guard cell morphogenesis than the *scap1* mutant. Functional analysis of MUS will shed light on the pathways controlling stomatal development.

## PATROL1 Affects Stomatal Movement and Plant Biomass via Controlling H^+^-ATPase Translocation to the PM

**Fig. 5:**
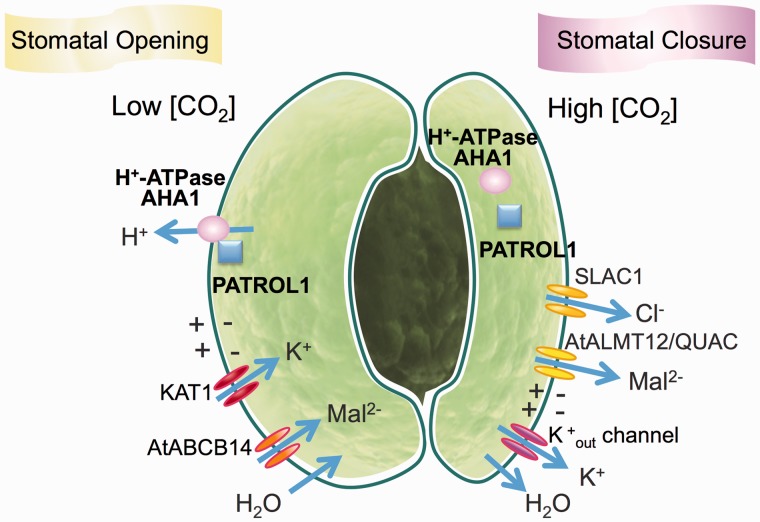
The intracellular localization of PATROL1 is controlled by [CO_2_] and affects the localization of AHA1. Low [CO_2_] promotes PATROL1 transfer to the close proximity of the PM, where PATROL1 facilitates the tethering of the H^+^-ATPase AHA1 to the PM. Under high [CO_2_], PATROL1 is translocated to the cytoplasm; as a result, it cannot promote the translocation of PM transporters.

Stomatal opening is initiated by the activation of the H^+^-ATPase in the guard cell PM. Because enhanced H^+^ extrusion by the H^+^-ATPase leads to hyperpolarization and increased K^+^ uptake, the guard cells swell and the stomata open. Blue light stimulates the H^+^-ATPases, through phosphorylation of their C-termini (Kinoshita and Hayashi 2011). The effects of [CO_2_] on stomatal movements are independent of light and photosynthesis, since in CO_2_-free air, plants fail to close their stomata in the dark (Heath and Russell 1954). It has been reported that low [CO_2_] triggers hyperpolarization, and that elevated [CO_2_] inhibits H^+^ efflux through the PM H^+^-ATPase (Edwards and Bowling 1985), but how CO_2_ affects the H^+^-ATPase remains unknown. A recent study shows that a Munc13-like protein, PATROL1 (PROTON ATPase TRANSLOCATION CONTROL 1), tethers H^+^-ATPase to the PM, contributing to the stomatal opening induced by low [CO_2_] (Hashimoto-Sugimoto et al. 2013). The Arabidopsis *patrol1* mutant isolated by thermal imaging exhibits increased leaf temperature even under low [CO_2_] (Hashimoto-Sugimoto et al. 2013). The *patrol1* mutant is impaired in stomatal opening in response to low [CO_2_] and light. The *PATROL1* gene encodes a protein with a MUN domain, which has been suggested to be required for intercellular membrane traffic in animal nerve cells by promoting the formation of soluble NSF attachment protein receptor (SNARE) complexes (Basu et al. 2005, Ma et al. 2011). Representative animal MUN domains exhibit weak amino acid identities with PATROL1. On the other hand, various higher plants have PATROL1 orthologs with highly conserved motifs distributed across the entire sequences, suggesting an essential function for these genes in higher plants (Hashimoto-Sugimoto et al. 2013). PATROL1 is expressed in whole plants including stomatal guard cells, and appears to be located in the endosome. Intriguingly, the intracellular distribution of PATROL1 depends on environmental conditions. PATROL1 is detected in close proximity to the PM under conditions that promote stomatal opening (well-watered plants in the light), while it is observed in the interior of the cell as numerous punctate structures under conditions that induces stomatal closure (darkness or desiccation) (Fig. 5). The loss-of-function mutation of *patrol1* disturbs the normal PM localization of Arabidopsis H^+^-ATPase AHA1, but does not affect the localization of the S-type anion channel SLAC1, the aquaporin PIP2a and the inward rectifying K^+^ channel KAT1 (Hashimoto-Sugimoto et al. 2013) (Fig. 5). Evidently, PATROL1 is target selective and has a role in tethering AHA1 to the PM during stomatal opening (Figs. 3, 5). The fungal phytotoxin FC induces stomatal opening by continuous activation of the PM H^+^-ATPases due to inhibition of their dephosphorylaiton. FC-induced stomatal opening is severely impaired in *patrol1* mutant plants (Hashimoto-Sugimoto et al. 2013), although at least 11 different PM H^+^-ATPases are expressed in Arabidopsis guard cells (Ueno et al. 2005). This suggests that not only AHA1 but also other H^+^-ATPases may be affected by PATROL1. *PATROL1*-overexpressing plants (PATROL1-OX) exhibit strong FC responses, indicating increased AHA levels in the PM of guard cells. The rise in stomatal conductance of PATROL1-OX in response to low [CO_2_] or light is rapid and enhanced compared with that of the WT. This leads to slightly higher CO_2_ assimilation rates, and results in increased biomass. *PATROL1*-overexpressing plants show a 32% increase in fresh weight compared with the WT when grown under short-day conditions (Hashimoto-Sugimoto et al. 2013).

Stomatal opening is often linked to enhanced CO_2_ assimilation rates; however, continuous stomatal opening (e.g. by constitutive activation of H^+^-ATPases, or in ABA-deficient mutants) often leads to small plant size and susceptibility to drought stress (Léon-Kloosterziel et al. 1996, Merlot et al. 2007). PATROL1-OX plants show normal stomatal closure in response to high [CO_2_], darkness and desiccation. The increased ability to prevent water loss may be a reason for the increased biomass production in PATROL1-OX. H^+^-ATPases acidify the apoplast and loosen the cell wall, which promotes cell expansion and growth according to the acid-growth theory (Hager 2003). Since *PATROL1* is expressed in the entire plant, increased amounts of H^+^-ATPase in the PM may also promote growth in PATROL1-OX. One may expect that overexpression of H^+^-ATPases enhances plant biomass as well, but this is not always the case due to the down-regulation of H^+^-ATPase activity (Zhao et al. 2000, Gévaudant et al. 2007, Haruta et al. 2010). Actually, overexpression of *AHA1* decreases biomass production, while overexpression of *PATROL1* increases it (Hashimoto-Sugimoto et al. 2013). These results suggest that PATROL1 is required for efficient biomass production by localizing the proper amount of H^+^-ATPase to the PM at the right time, without down-regulating its activity. Highly conserved sequences of *PATROL1* genes observed in numerous higher plants suggest the possibility of increasing the biomass of food crops and other naturel resources.

Membrane traffic by endo- and exocytosis affects stomatal movement through changes in the density of ion transporters and the PM surface area (Shope et al. 2003, Grefen et al. 2011). SNARE proteins are essential components for intracellular vesicle fusion during membrane traffic. The SNARE protein SYP121 in tobacco has been identified as an ABA-related signaling component, and the disruption of SYP121 function prevented the ABA-induced inhibition of 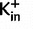 channels (Leyman et al. 1999). Sutter et al. (2006) showed that Sp2 fragments of tobacco and Arabidopsis SYP121 disrupted the mobility and delivery of the 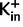 channel KAT1, but not that of the H^+^-ATPase PMA2 to the PM, indicating selectivity of SNARE-mediated traffic to the PM. The loss-of-function *syp121* mutant in Arabidopsis exhibits delayed stomatal opening and develops similar or smaller rosettes than WT plants, depending on the environmental conditions (Eisenach et al. 2012). After stomatal closure, reopening of stomata is delayed in *syp121*, correlating with the slow recovery of KAT1 traffic and recycling to the PM (Eisenach et al. 2012). These results indicate that the control of the amount of K^+^ channels in the PM by SNARE proteins is important for stomatal movement and plant growth. Transport of H^+^-ATPases to the PM is not a default process but requires cytosolic domains; the underlying mechanisms have not been clarified yet (Lefebvre et al. 2004). In kidney cells in animals, the recycling of H^+^-ATPases to the PM by membrane traffic is an important mechanism for controlling H^+^ secretion. The process is mediated by the SNARE protein syntaxin1 which binds to distinct regions of H^+^-ATPases (Schwartz et al. 2007). PATROL1 affects the localization of AHA1 but not that of KAT1 (Hashimoto-Sugimoto et al. 2013), suggesting that SNARE proteins which potentially associate with PATROL1 and AHA1 may not include SYP121. PATROL1 may control the amount of H^+^-ATPases in the PM by regulating the formation of the specific SNARE complexes that include SNARE proteins which associate directly with the H^+^-ATPases.

ROP GTPases function as molecular switches in diverse cellular process. One of the ROP GTPases, ROP2, is a negative regulator of stomatal responses to light, ABA and CO_2_ (Jeon et al. 2008, Hwang et al. 2011). Light induces the activation of ROP2 and its translocation to the PM, whereas ABA induces its inactivation and translocation to the cytoplasm. Constitutively active ROP2, mainly located on the PM, inhibits stomatal closure by suppressing the internalization of the PM in guard cells (Hwang et al. 2011). This mechanism allows for finely controlled stomatal apertures by regulating endocytotic membrane trafficking.

## Concluding Remarks

A doubling of ambient CO_2_ levels, as is expected to occur within the next century, has been predicted to reduce stomatal conductance by as much as 40% (Morison 1987). Given the potentially large impact of this effect on plant water status and atmospheric conditions, it would be of interest to elucidate the physiological basis of the CO_2_-sensing and response mechanisms in guard cells. Recent studies have advanced the understanding of CO_2_ signaling mechanisms, but the number of identified genes involved in these processes remains limited. For example, a CO_2_ sensor, which directly binds CO_2_ and whose loss completely abolishes stomatal CO_2_ responses, has not been isolated yet. The CO_2_ sensor may bind to 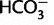 and activate HT1 kinase. Stomatal conductance responds to intercellular [CO_2_] rather than to [CO_2_] on the leaf surface (Mott 1988). Stomata in isolated epidermal strips respond to [CO_2_] changes, indicating that the CO_2_ sensor is associated with the guard cells (Webb et al. 1996, Brearley et al. 1997). However, the responses observed in isolated epidermis are much smaller than those seen in leaves. Mott et al. (2008) showed that stomata in isolated epidermis of *Tradescantia pallida* and *Pisum sativum* develop limited responses to CO_2_, whereas stomata in epidermal strips placed on a mesophyll layer respond rapidly and reversibly to CO_2_. Thus, signals from the mesophyll are controlling stomatal responses. The signaling from the mesophyll is inhibited by polyethylene spacers inserted between the epidermal layer and mesophyll; cellophane spacers have no such effect (Fujita et al. 2013). This indicates that mesophyll-derived signals are small molecules dissolved in the liquid. Mesophyll-derived malate, which plays an important role in regulating stomatal movements (Hedrich et al. 1994, Araújo et al. 2011), may be a candidate signaling molecule. The identification of the mesophyll-derived signal molecules will provide essential information about CO_2_ signaling mechanisms including guard cell receptors that sense CO_2_ signals from the mesophyll.

Recent studies in guard cells have identified important genes and molecules involved in stomatal responses. Screening of mutants provides a powerful means to identify novel signaling components, because this approach might produce what nobody expects. CO_2_ sensors in guard cells and/or mesophyll cells may be identified by forward genetic approaches in the future.

## Funding

This work was supported in part the Ministry of Education, Science and Culture of Japan [Grants-in-Aid (21114002 to K.I., 22570045 to K.K., 25891020 to J.N.); the Fumi Yamamura Memorial Foundation for Female Natural Scientists [to M. H.-S.].

## Disclosures

The authors have no conflicts of interest to declare.

## Abbreviations

AHA1: ARABIDOPSIS H^+^-ATPase 1
CA: carbonic anhydrase
FC: fusicoccin
HT1: HIGH LEAF TEMPERATURE 1
MAPK: mitogen-activated protein kinase
OST1: OPEN STOMATA 1
PATROL1: PROTON ATPase TRANSLOCATION CONTROL 1
PM: plasma membrane
SCAP1: STOMATAL CARPENTER 1
SLAC1: SLOW ANION CHANNEL 1
SNARE: soluble NSF attachment protein receptor
WT: wild type
